# Supplementary material for: Extension of the Coherence Time by Generating MW Dressed States in a Single NV Centre in Diamond
Source: Sci Rep. 2019 Sep 16;9:13318. doi: 10.1038/s41598-019-49683-z (PMC6746786; doi:10.1038/s41598-019-49683-z)
Supplement: Supplementary file 1 — Supplementary Information: Extension of the Coherence Time by Generating MW Dressed States in a Single NV Centre in Diamond [file 41598_2019_49683_MOESM1_ESM.pdf]

Supplementary Information: Extension of the Coherence Time by Generating MW Dressed States in a Single NV Centre in Diamond

H. Morishita,[1,2] T. Tashima,[1,3] D. Mima,[2] H. Kato,[4] T. Makino,[4] S. Yamasaki,[4] M. Fujiwara,[2] and N. Mizuochi[2]

1 These authors equally contributed to this work.

2 Institute for Chemical Research, Kyoto University, 611- 0011, Japan

3 Department of Electronic Science and Engineering, Kyoto University, 615-8510 Kyoto, Japan

4 Energy Technology Research Institute, National Institute of Advanced Industrial Science and Technology (AIST), Ibaraki 305-8568, Japan

Estimation of the sensitivity of an NV magnetometer with mw dressed states

We estimate the sensitivity of a quantum sensor with mw dressed states in the case of sensing for weak AC magnetic field. The key of our sensing is that the sensing information of the AC magnetic field in the mw dressed states can be adding. By using the fact that the dressed states have the same resonant frequencies, the number of dressed states generated for our sensing are then used in order to boost up the sensitivity of the target. Namely, we can add the information corrected at the same time using a quantum adder [S1]. Figure S1(a) shows the sequence to demonstrate the quantum sensing with the dressed states for the weak AC magnetic field. This sequence is considered as a combination of generation of dressed states and conventional weak AC magnetic field sensing with a Hahn echo sequence described in Fig. S1(b) [S2]. The details of the sequence described in Fig. S1(a) are followings: after the initialisation of an NV electron spin by a pulse laser, applications of a first $\pi/2$ pulse and pulsed strong mw-driving field generate dressed states in the NV centre. Under the generation of the dressed states, the sensing information of the AC magnetic field is stored in these states by using the Hahn echo sequence. Finally, the stored information shown in Fig. S2 is corrected by a quantum adder. The quantum adder consists of a Hadamard gate H and a unitary operator U^n of $|\Psi\rangle$. The $|\Psi\rangle$ works as an ancilla state, and the sensing information in n dressed states ($|\phi_n\rangle$) is transferred to $|\Psi\rangle$. For example, $|-1,0\rangle$ of an NV centre can be used for an ancilla state under the irradiation of drive field between $|-1,1\rangle$ and $|0,1\rangle$ of the NV centre described in Fig. S1(a) in the main text.

A minimum detectable value of the magnetic sensor ($B_{\rm min}$) is given by the following relation: $B_{\rm min} \propto \frac{1}{\sqrt{NT_2}}$, where N and T_2 are number of NV centre (number of qubits) and T_2 of NV electron spin [S2]. In the case of magnetometry with a single NV centre, N = 1. In our experiments, we observed $T_{2\rm p} \approx 1.5$ ms and $T_2 \approx 4.2$ μs with and without the generation of dressed states in Fig. 6 of the main paper. It is noted that at least four dressed states were generated in the $T_{2\rm p}$ measurement. In this case, we numerically confirm that the sensitivity is approximately enhanced by 27 times using these values and this relation with $N = M$ when $2M$ virtual states can be prepared by an irradiation of a strong mw-driving field.

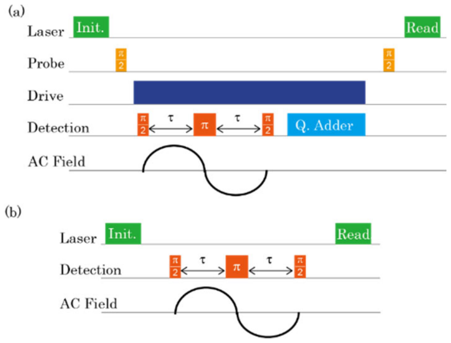

FIG. S1. Pulse sequence to demonstrate AC magnetic field sensing (a) with and (b) without virtual quantum states.

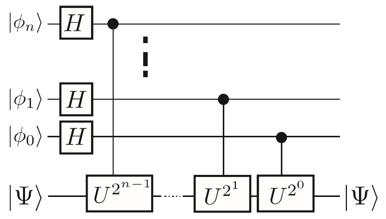

FIG. S2. Quantum circuit of the quantum adder.

[S1] Draper, T. G. Addition on a Quantum Computer. *arXiv* Preprint at https://arxiv.org/abs/quantph/0008033 (2000).

[S2] Taylor, J. M., *et al.*, High-sensitivity diamond magnetometer with nanoscale resolution. *Nat. Phys.* **4**, 810 (2008).
